# War Patients at the Hospital

**Published:** 1914-09-05

**Authors:** Ernest W. Morris

**Affiliations:** the Secretary


### PREPARATION AND RECEPTION OF SOLDIER PATIENTS AT THE LONDON HOSPITAL.

### Special Report by Mr. ERNEST W. MORRIS, the Secretary.

The Chairman during the first days of the war offered 500 beds to the Government—250 to the Army and 250 to the Navy—for sick and wounded men. Both offers were officially accepted. The Secretary, on his return to London owing to the Chairman's departure through illness and overwork, approached the War Office to discover whether there was any likelihood of the beds being used at an early date, or indeed at all. He was informed that it was extremely likely that they would be used, but not for some weeks. That the military hospitals would be used first, then the Territorial hospitals, and then the voluntary hospitals. On this information it was decided to begin to fill up the empty beds again with short-time cases, as their disuse was likely to cause considerable suffering amongst the poor in the East End, suffering which was likely to increase as time went on. That food was becoming more normal in price was an important factor in coming to this decision. The staff were requested, therefore, to admit cases, and especially those who required surgical attention to fit them for the King's Service. Lists were circulated to the staff, however, warning them to abstain as far as possible from ordering certain drugs of which it was impossible to obtain further supplies after the consumption of our present stock.

### Who Should Treat the Wounded?

A letter was recently received from Colonel Skinner of the Horse Guards asking whether this hospital would allow the 250 beds it had offered to become a section of No. 2 London General Territorial Hospital. As such an amalgamation seemed to move the hospital one step nearer the firing line it seemed advisable to agree if proper conditions were offered, and accordingly the Secretary visited Colonel Skinner, who is in charge of the sick-bed arrangement in London, in order to discuss the question. Three or four interviews took place. Colonel Skinner wished, amongst other things, that only officers of the Territorial Hospital should be allowed to treat the soldiers here, and that the beds allotted to wounded soldiers should, as far as possible, be in a ringed fence, forming practically a military hospital within the hospital. The Secretary insisted on the principle that London Hospital patients could only be treated by the London Hospital staff, and at one of the interviews suggested that all the London Hospital staff should be given a temporary commission during the war, and thus each would be able to treat military cases. He considered that this should apply even to the resident house physicians and house surgeons, all of whom were willing to waive all question of pay to which they might be entitled. Colonel Skinner received this suggestion not unsympathetically, and stated that he would consider it.

### Wounded to Arrive on Sunday.

On Friday, August 28, at an interview, the Secretary was informed that cases were not likely to arrive for five or six weeks, and that when they did come the hospital would receive plenty of notice. The Admiralty also informed us that we should have at least twenty-four hours' notice by wireless before any of their cases arrived. On Sunday last, however, a message was received in the morning asking us whether we could admit 100 wounded men that evening. We willingly agreed to do so.

Captain Fenwick, one of our consulting staff, well known to all the governors as Mr. Hurry Fenwick, visited Colonel Skinner during Sunday morning with the Secretary, and Colonel Skinner asked Captain Fenwick to fill the office of Military Superintendent at the hospital, and so to act as a go-between between the War Office and the hospital. Captain Fenwick agreed to supply the War Office with all necessary forms and information, and to act generally at the hospital as the military representative of Colonel Skinner, subject to the approval of this Court. The Secretary stated that this arrangement is likely to be of the greatest possible assistance to him as, instead of having to communicate with various members of the War Office staff in matters connected with military law, he can communicate direct with Captain Fenwick.

### Preparations and Arrival.

"We were informed that the wounded troops would arrive on Sunday evening about 7 o'clock, and your Secretary and Matron and one of Matron's assistants, Miss Monk, arranged for the provision of extra beds, and Matron made the necessary arrangement with regard to the nursing staff. During the afternoon we were asked by the War Office by telephone if we could arrange for the transport of the wounded from Waterloo to the hospital. This, through the invaluable help of a member of the committee—Mr. Alfred Salmon—we were fortunately able to do."

The Secretary gratefully acknowledges the courtesy received from a sister hospital, the West London. "The West London," he stated, "was quite as anxious to have the honour of receiving the wounded as we were. Nevertheless, when they knew that the honour was to come to us they exerted themselves to provide and to lend to us at a most inconvenient time (Sunday afternoon) thirty mattresses for carrying the wounded to us."

### All Admitted about 9 p.m.

"The hospital also arranged that a large party of its residents and students should go to Waterloo to act as stretcher-bearers and carriers under Capt. Fenwick's orders, who agreed to be there. The wounded men, of whom there were exactly 100, were safely admitted to the hospital about 9 p.m. on Sunday night, without hitch or difficulty, thanks to the excellence of the voluntary help. On account of the enormous crowds which had formed in Whitechapel Road, we admitted the soldiers by the back gate, and they were driven up to the steps facing the Queen's statue. A staff of police were kept in readiness to deal with the crowd should they become unmanageable at the back gates."

### A False Alarm.

"While the admission of these soldiers was actually proceeding a further message was received from the War Office, asking us to admit 150 more wounded during the night—they might arrive at 5 a.m.—and we were again asked to supply transport, stretchers, and carriers. Matron was informed of this latter demand, and sent word to the Secretary that she would devise a scheme for the admission of these new cases while he continued the superintendence of the cases then arriving. The War Office, however, telephoned that the soldiers would not arrive until 10 o'clock. The patients duly arrived between 10 and 11, and by similar arrangements as were adopted on the previous day 200 men were safely admitted into the beds; the total, therefore, of 300 in all."

The Secretary wished to express his own personal indebtedness, as well as that of the hospital, to the Matron and to her assistants, especially to Miss Monk. "Matron," he said, "was equally ready to help at midnight or mid-day, and that these men were so easily and quickly put into our wards, and necessary nurses promptly provided, was due to her ingenuity and wisdom. It would have been impossible to carry out this undertaking but for her forethought and untiring assistance. I count it a privilege to say this, and that without her our difficulties would have been quite unsurmountable."

### Some Loyal Service.

The Secretary testifies to the willing and ungrudging help given by every nurse in the hospital. All nurses asked to be allowed to stay on duty until midnight on Sunday, in order to see that all patients were comfortable. He refers to the help given by the residents, who co-operated heartily in assisting us to provide the necessary beds, and to the students who live in the hostel for volunteering on both occasions to act as assistants and stretcher-bearers between Waterloo and the hospital. The steward spent most of the day and night in arranging for the catering of this unexpected addition to his numbers; the cook, who had left the hospital, was sent for, and returned in order to have large quantities of beef-tea ready; and the milk contractors sent 25 gallons of milk by special messenger, so that there was at least a quart for each man on arrival.

The porters worked night and day, regardless of hours, in moving patients from one ward to others, and in carrying patients on their arrival to their wards. Fifty more patients arrived in the second batch than were expected. The police were present in large force, and, as always, gave invaluable help.

### The Thanks of the War Office.

The medical and surgical staff who were on "full duty" at the time, had been previously notified that the wounded were expected, and were here to receive them. Some of the staff were at work during most of the night and the following day. The War Office, through Captain Fenwick, who had been at the hospital almost night and day since Sunday morning, altogether neglecting his own comfort and his heavy private practice, has sent a very complimentary message to the hospital. That the hospital should have merited any such commendation is due to the fact that every man and woman in it, from committee-man to lift-boy, did his duty very heartily as the wounded men had done. The men have been allowed to smoke in the wards, and this has been a great delight to them, and many presents of pipes, tobacco, and cigarettes have been received. We have had valuable gifts of clothing, etc., from Queen Mary's Needlework Guild and many other societies; the Red Cross Society in the Borough of Camberwell sent a most valuable present on Tuesday of about 100 shirts, nearly 100 flannel jackets, 70 pyjama suits, socks, etc. Some of the great newspaper proprietors are sending large supplies of newspapers for distribution in the wards.

